# Alterations of brain microstructures in a mouse model of prenatal opioid exposure detected by diffusion MRI

**DOI:** 10.1038/s41598-022-21416-9

**Published:** 2022-10-12

**Authors:** Gregory G. Grecco, Syed Salman Shahid, Brady K. Atwood, Yu-Chien Wu

**Affiliations:** 1 Department of Pharmacology and Toxicology, Indiana University School of Medicine, Indianapolis, IN 46202 USA; 2 Indiana University School of Medicine, Medical Scientist Training Program, Indianapolis, IN 46202 USA; 3 Department of Radiology and Imaging Sciences, Indiana University School of Medicine, 355 West 16th Street, Suite 4100, Indianapolis, IN 46202 USA; 4 Stark Neurosciences Research Institute, Indiana University School of Medicine, Indianapolis, IN 46202 USA

**Keywords:** Neuroscience, Biomarkers, Prognostic markers

## Abstract

Growing opioid use among pregnant women is fueling a crisis of infants born with prenatal opioid exposure. A large body of research has been devoted to studying the management of opioid withdrawal during the neonatal period in these infants, but less substantive work has explored the long-term impact of prenatal opioid exposure on neurodevelopment. Using a translationally relevant mouse model of prenatal methadone exposure (PME), the aim of the study is to investigate the cerebral microstructural differences between the mice with PME and prenatal saline exposure (PSE). The brains of eight-week-old male offspring with either PME (n = 15) or PSE (n = 15) were imaged using high resolution in-vivo diffusion magnetic resonance imaging on a 9.4 Tesla small animal scanner. Brain microstructure was characterized using diffusion tensor imaging (DTI) and Bingham neurite orientation dispersion and density imaging (Bingham-NODDI). Voxel-based analysis (VBA) was performed using the calculated microstructural parametric maps. The VBA showed significant (*p* < 0.05) bilateral alterations in fractional anisotropy (FA), mean diffusivity (MD), axial diffusivity (AD), radial diffusivity (RD), orientation dispersion index (ODI) and dispersion anisotropy index (DAI) across several cortical and subcortical regions, compared to PSE. Particularly, in PME offspring, FA, MD and AD were significantly higher in the hippocampus, dorsal amygdala, thalamus, septal nuclei, dorsal striatum and nucleus accumbens. These DTI-based results suggest widespread bilateral microstructural alterations across cortical and subcortical regions in PME offspring. Consistent with the observations in DTI, Bingham-NODDI derived ODI exhibited significant reduction in PME offspring within the hippocampus, dorsal striatum and cortex. NODDI-based results further suggest reduction in dendritic arborization in PME offspring across multiple cortical and subcortical regions. To our best knowledge, this is the first study of prenatal opioid exposure to examine microstructural organization in vivo. Our findings demonstrate perturbed microstructural complexity in cortical and subcortical regions persisting into early adulthood which could interfere with critical neurodevelopmental processes in individuals with prenatal opioid exposure.

## Introduction

The opioid addiction epidemic has led to a significant rise in maternal opioid use disorder which has ultimately contributed to a subsequent crisis in prenatal opioid exposure^1^. Opioids used during pregnancy are able to cross the placenta and produce neonatal abstinence syndrome (also referred to as neonatal opioid withdrawal syndrome) in the newborn which is characterized by symptoms of tachycardia, hyperthermia, sweating, GI disturbances, changes in feeding patterns, sleeping issues, and hyperirritability^2^. Rates of neonatal abstinence syndrome have risen 82% in the U.S. between 2010 and 2017^1^. While the characterization and pharmacological management of neonatal abstinence syndrome has been fairly well described, the long-term impact of prenatal opioid exposure on neurodevelopment remains to be fully explored.

Clinical neuroimaging studies of infants and children with prenatal opioid exposure have discovered several differences in brain macrostructure^3–5^, morphometry^6–10^, microstructure^11–13^, and functional connectivity^14–16^. For instance, infants with prenatal opioid exposure may have decreased brain volumes in multiple regions including the bilateral ventrolateral nuclei of thalamus, bilateral subthalamic nuclei, bilateral insular white matter, and brainstem^5^. Additionally, Radhakrishnan et al., identified increased functional connectivity between the right amygdala and medial prefrontal cortex in infants with prenatal opioid exposure^14^. For a more extensive review of human neuroimaging studies in prenatal opioid exposure, please see Radhakrishnan et al., 2021^17^. Unfortunately, clinical neuroimaging studies are often small, and few studies are able to control for potential confounding variables, such as smoking, polysubstance use, co-morbid health conditions, or low birth weights that are present at higher rates in this population and could impact neurodevelopment^18–20^. Hence, animal models provide an opportunity to examine the effects of prenatal opioid exposure on neurodevelopment in the absence of confounding variables present in the maternal/fetal environment.

Our laboratory has developed a mouse model of prenatal exposure to methadone^21^ which is an opioid analgesic and that has been widely used in the treatment of opioid use disorder for decades^22^. Because methadone is a gold-standard treatment for opioid addiction, prenatal methadone exposure (PME) represents a significant proportion of all opioid exposure cases in infants^23,24^. We have discovered that PME disrupts early neurobehavioral developmental milestones, produces hyperactivity, and alters reward-related behavior in mouse offspring^21,25^. Additionally, offspring demonstrate altered neuronal excitability and synaptic signaling in the motor cortex, somatosensory cortex, and the dorsal striatum (analogous to the human caudate/putamen) that persists into early adolescence which is likely to contribute to the aberrant behavioral development in PME offspring^21,25–27^*.*

Preclinical neuroimaging studies of prenatal opioid exposure have been very limited. To our knowledge, only three small animal neuroimaging studies have been completed with one performing ex vivo diffusion tensor imaging (DTI) of two major white matter tracts^28^ and one employing single voxel magnetic resonance spectroscopy (MRS)^29^. Previously, we examined several regions of interest for macroscopic differences using in vivo ultra-high-field structural MRI in PME offspring but did not identify any differences in macrostructure compared to controls^21^. Similarly, histological analyses revealed PME had very limited effects on cortical laminations in the anterior cingulate cortex, motor cortex and somatosensory cortex suggesting gross brain structure in offspring remains mostly intact^21^. However, as opioids are not canonically considered a neurotoxic or teratogenic agent, the effects of PME on the brain are likely more subtle which may present as microstructural tissue differences only sensitive to more advanced neuroimaging techniques.

Diffusion MRI (dMRI) can provide meaningful insight into the microstructural organization of a tissue. The application of dMRI derived signal representation schemes or tissue-specific models may provide information on the subtle microstructural changes associated with prenatal drug exposure. DTI which estimates water-self diffusion in a tissue microenvironment as a three-dimensional Gaussian model has been used extensively to quantify microstructural changes in brain due to aging and under various pathological conditions^30–36^. Even though DTI is highly sensitive to microstructural alterations, due to its non-specific nature, it is challenging to associate those changes in diffusivity profile to any specific tissue features. Biophysically inspired models of diffusion attenuated signal estimate non-Gaussian diffusion behavior and have shown to provide additional and complementary information to DTI^37–41^. Neurite orientation dispersion and density imaging (NODDI) is a biophysically inspired model of diffusion that aims to provide more specificity to water diffusion attenuated signal in cerebral microstructural environment^42^. The original NODDI model is based on Watson distribution to map dispersion pattern, however it is insensitive to anisotropic orientation dispersion thus cannot estimate anisotropy in neurite organization^43^. The advanced Bingham-NODDI model used in this study can provide information related to dispersion anisotropy and thus may facilitate our understanding on neurite organization in the brain tissue. We hypothesized that the sensitivity of DTI and specificity of the Bingham-NODDI model may uncover changes in tissue microstructures in the brain of PME offspring which, otherwise, could not be observed using conventional structural MRI. In the present study, we employed a voxel-based statistical analysis to compare the DTI and NODDI derived parametric maps in male offspring at eight-weeks of age, an age at which the mouse brain has reached full maturity, with either PME or prenatal saline exposure (PSE)^44^.

## Materials and methods

### Animal preparation

**Figure 1:**
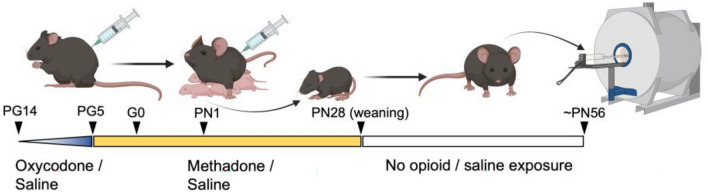
Diagram of experimental timeline. Adult female C57Bl/6 J mice are treated with an escalating dose of oxycodone or saline for 9 days prior to mating for pregestational (PG) exposure. Five days prior to mating (PG day 5) the female mice were switched to a maintenance dose of methadone or continued on with saline exposure. The female mice continued to receive methadone or saline from the day of mating/start of gestation (G0) throughout pregnancy. Methadone and saline were also administered to the mothers during the offspring’s postnatal period from postnatal day 1 (PN1) to PN28 upon which the offspring were weaned. Offspring received no direct administration of opioids or saline during the neonatal period or after weaning. Around 8 weeks of age, male offspring underwent imaging.

All animal care and research were conducted within the guidelines established by the National Institutes of Health, and all our protocols were approved by the Indiana University School of Medicine Institutional Animal Care and Use Committee and the reporting in the manuscript follows the recommendations in the ARRIVE guidelines^45^. The full characterization and description of this model has been published previously^21^. A schematic of the timeline is presented in Fig. 1. Single-housed female C57BL/6 J mice were randomly assigned to receive either saline (10 mL/kg) or a dose-ramping schedule of oxycodone (a frequently misused prescription opioid analgesic) for nine days to model oxycodone dependence pregestationally (ramping from 10 mg/kg up to 30 mg/kg). All saline or oxycodone doses were administered subcutaneously twice daily at least seven hours apart. Following nine days of oxycodone injections, oxycodone-dependent mice began receiving methadone (10 mg/kg s.c. b.i.d.) to model treatment for opioid use disorder while saline-treated animals continued to receive saline injections. After five consecutive days of methadone treatment, an 8-week-old C57BL/6 J male mouse was placed into the cage of each female for four days to mate. All methadone or saline treatments to the mother continued throughout mating, the remainder of pregnancy, and the postnatal period up to weaning at approximately postnatal day 28. We have characterized opioid levels in the mother and offspring brain and plasma at various intervals prenatally and postnatally and demonstrated that this dose of methadone leads to plasma levels within the human therapeutic range^21^. Furthermore, the dosing strategy induces symptoms of opioid dependency in both dams and offspring, yet maternal care behaviors remained unaffected by opioid treatment^21^. Oxycodone and methadone were obtained from the National Institute on Drug Abuse Drug Supply Program. For the following imaging studies, no more than two offspring were used per litter to minimize litter effects and mice were housed 2–4 per cage until imaging was completed at approximately 8–9 weeks of age.

### Diffusion MRI acquisition

The diffusion weighted images (DWIs) were acquired in a horizontal bore 9.4 Biospec pre-clinical MRI system (Bruker BioSpin MRI GmbH, Germany) equipped with shielded gradients (maximum gradient strength = 660 mT/m, rise time = 4570 T/m/s) and ^1^H cryogenic surface coil (Cryoprobe, Bruker, BioSpin). A 2D multi-shell diffusion acquisition scheme was used. DWIs were acquired using a multi-shot dual-spin-echo echo planar imaging (EPI) sequence using the following parameters: TE/TR = 35.84/3000 ms, δ/Δ = 3/15 ms, matrix size = 128 × 125, voxel size = 130 × 130x130 µm^3^, number of slices = 40, slice thickness = 130 µm, 2 b-value shells (1000 and 2000) s/mm^2^, 10 b0 (5 for each shell), 32 diffusion encoding direction for b = 1000 and 56 for b = 2000 s/mm^2^. For MRI scan, initially mice were anesthetized under 3% isoflurane in an induction chamber. The anesthetized mice were transferred to an MR compatible cradle and positioned in an MRI compatible head holder to minimize head motion. Anesthesia was then maintained at 1.5% isoflurane in 100% oxygen throughout imaging. Respiration rate was monitored using a pressure pad placed under the animal abdomen and animal body temperature was maintained by a warming pad (37 °C) placed under the animal.

### dMRI data processing and model fitting

The raw magnitude DWI data was preprocessed to reduce the effect of noise using the Non-Local Means (NLMeans) filter implemented in Anima (https://anima.irisa.fr)^46^ Using the first b0 image from the denoised data, initial brain mask was obtained in a semi-automated way using Rapid Automatic Tissue Segmentation algorithm (RATS)^47^. The skull-stripped brain mask from the b0 image was used to isolate brain region in the rest of the DWIs. Motion and Eddy current induced geometric distortions were corrected from the skull-stripped denoised DWI data using the ‘*eddy_correct*’ functions in FMRIB Software Library (FSL v6.0)^48^. For B1 field inhomogeneity correction, ‘*N4BiasField’* correction algorithm implemented in MRtrix3 was used^49^.

**Figure 2:**
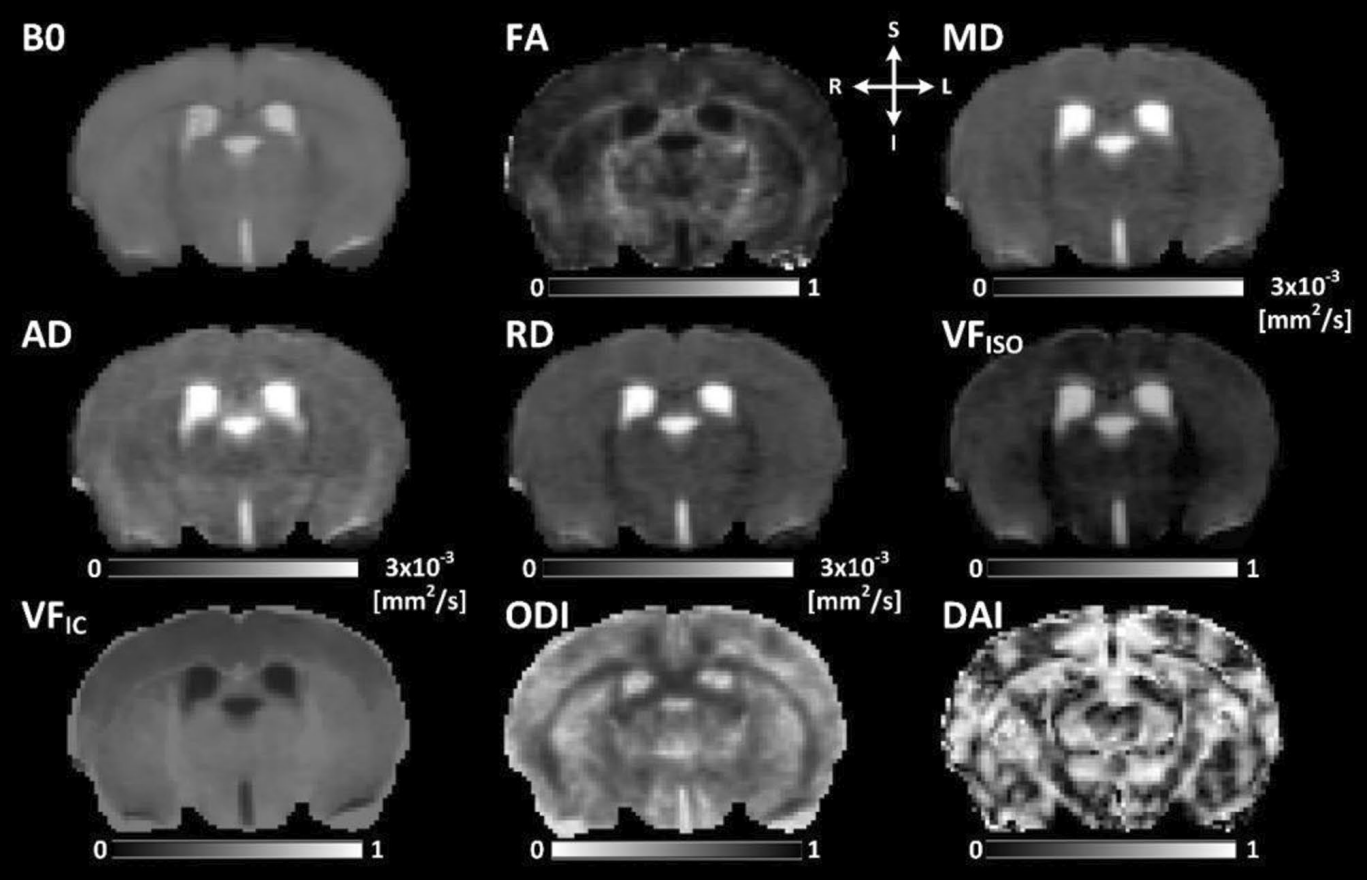
Microstructural parametric maps derived from DTI and NODDI using one of the PSE animals. L and R indicate anatomical orientation: left–right. S and I indicate anatomical orientation: superior–inferior. B0: non-diffusion weighted image, FA: fractional anisotropy, MD: mean diffusivity, AD: axial diffusivity and RD: radial diffusivity maps, were derived from DTI. VF_ISO_: volume fraction of isotropic water diffusivity, VF_IC_: intracellular volume fraction, ODI: orientation dispersion index and DAI: dispersion anisotropy index, were derived from the Bingham-NODDI model.

DTI metrics were estimated using the pre-processed DWI data from b = 0 and 1000 s/mm^2^. The voxel based DTI fitting was performed using ‘*DTIFIT*’ function in FSL library^50^. Parametric maps of fractional anisotropy (FA), mean diffusivity (MD), axial diffusivity (AD) and radial diffusivity (RD) were derived from the fitted DTI model. Multi-compartment microstructural imaging was performed using Bingham-NODDI, which has been shown to be more specific to the underlying microstructure and is capable of estimating the anisotropic orientation dispersion of neurites^43^. To achieve a more physiologically plausible representation of the tissue microstructure^51^, the model R^2^ coefficient of determinations (i.e., the goodness of fit) were evaluated as a function of the initial condition, the intrinsic parallel diffusivity (D_||_), over the range from [0.6–1.7] × 10^–3^ mm^2^/s with a step size of 0.1 × 10^–3^ mm^2^/s. The mean values of R^2^ across our regions of interest including striatum, hippocampus and white matter were evaluated to determine a best initial condition for the model fitting. On average, the D_||_= 1.1 × 10^–3^ mm^2^/s produced the best fitting (i.e., highest R^2^) and was used in this study. NODDI-derived metrics, namely: orientation dispersion index (ODI), dispersion anisotropy index (DAI), volume fraction of isotropic water diffusivity (VF_ISO_) and intracellular volume fraction (VF_IC_) were calculated using the Dimpy toolbox, which is a python-based open-source software package^52^. Figure 2 shows the diffusion metrices and their quantitative range.

In order to assess the voxel-wise group differences between PME and controls, voxel-based analysis was used^53^. In brief, each parametric map derived from DTI and NODDI was used to construct respective study specific templates using ANTs ‘buildtemplateparallel.sh’ script^54^. In the next step each parametric map in subject space was nonlinearly registered to the respective (parametric) study specific template using ANTs ‘antsRegistrationSyN.sh’ script^54^. Subsequently, each registered parametric map was smoothed with a gaussian kernel (sigma = 3 voxels) for spatial smoothing. These registered and smoothed diffusion maps of each animal were stacked in the form of 4D stack of 3D volumes for voxel-wise statistical analyses.

### Statistical analysis

General linear model analysis was performed voxel-by-voxel for comparing the diffusion metrics between the PME and control group. Permutation tests were conducted to control for family-wise errors associated with multiple comparisons using ‘randomise’ functions in FSL library with 5000 permutations^55^. Mass-based Threshold-free cluster enhancement (TFCE) with family-wise error corrections for multiple comparisons was used to detect statistical difference at the cluster level with *p* < 0.05 (two-tailed)^56^.

## Results

**Figure 3:**
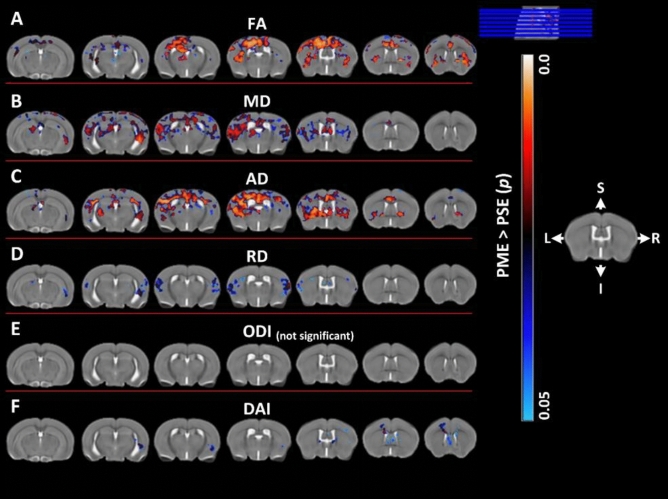
Voxel-wise statistical maps using DTI and NODDI derived parametric maps. Clusters depicted in false color indicate regions where PME (n = 15) animals exhibited significantly higher values compared to PSE (n = 15). The results were family-wise error corrected for multiple comparisons (*p* < 0.05). Variation in color maps highlight the degree of significance. The results are overlayed on a study specific B0 template for better visualization. FA: fractional anisotropy, MD: mean diffusivity, AD: axial diffusivity, RD: radial diffusivity, ODI: orientation dispersion index and DAI, dispersion anisotropy index. L and R indicate anatomical orientation: Left–Right. S and I indicate anatomical orientation: superior–inferior.

Voxel-wise analysis revealed several differences in DTI metrics indicating widespread changes to microstructure in PME offspring. Numerous clusters of increased FA were identified in PME offspring relative to PSE controls across various subcortical and cortical structures bilaterally (Fig. 3A). In more posterior regions, these clusters corresponded to portions of the retrosplenial, visual, and auditory cortex (primarily left-sided) that included some extension into the external capsule, cingulum, and corpus callosal white matter bilaterally (Fig. 3A). Subcortically, significant clusters were identified in the bilateral hippocampi, thalamic nuclei, habenula, the amygdalostriatal transition area and white matter of the dorsal fornix (Fig. 3A). Moving anteriorly, PME exhibited increased FA extensively in the somatosensory, motor, and cingulate cortex with some extension into the piriform and insular cortex bilaterally (Fig. 3). Significant clusters were also identified in the bilateral basal ganglia, including the caudate/putamen (i.e., dorsal striatum), globus pallidus and nucleus accumbens, as well as the adjacent internal capsule white matter and the claustrum (Fig. 3A). No significant clusters were identified where FA was reduced in PME offspring. MD and AD were also significantly increased in PME mice among many of the same regions where FA was significantly higher (Fig. 3B–C). PME offspring also displayed increased MD in the bilateral entorhinal and posterior insular cortex. Clusters of significantly increased MD and AD were also recognized in several other limbic structures including portions of the hippocampus, habenula, dorsal amygdala, septal nuclei, fimbria of the hippocampus, and stria terminalis bilaterally. RD was less impacted by PME than FA, MD, or AD (Fig. 3D). PME only demonstrated increased RD bilaterally in the external capsule, auditory, posterior insular, and somatosensory cortex, and smaller clusters within the hippocampus, dorsal striatum, septal nuclei, fornix, right claustrum, and the right dorsal amygdala (Fig. 3D).

**Figure 4:**
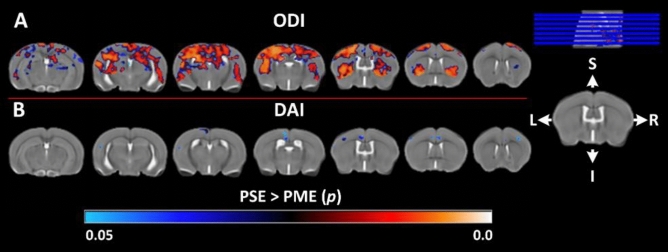
Voxel-wise statistical maps using NODDI derived parametric maps. Clusters depicted in false color indicate regions where PSE (n = 15) animals exhibited significantly higher values compared to PME (n = 15). The results were family-wise error corrected for multiple comparisons (*p* < 0.05). Variation in color maps highlight the degree of significance. The results are overlayed on a study specific b0 template (a T2W template) for better visualization. ODI: orientation dispersion index and DAI, dispersion anisotropy index. L and R indicate anatomical orientation: Left–Right. S and I indicate anatomical orientation: superior–inferior.

Neurite organization in PME offspring was also significantly altered in several cortical and subcortical structures throughout the brain (Figs. 3 and 4). PME revealed several significant clusters of altered DAI (Figs. 3 and 4). Particularly, in the posterior slices, DAI was significantly higher in the left claustrum, left dorsal amygdalostriatal transition zone, left somatosensory cortex, left external capsule, and left fornix (Fig. 3F). More anteriorly, significant clusters of increased DAI were identified within the white matter of the stria terminalis and internal capsule of PME offspring (Fig. 3F). In the most anterior regions, DAI was increased in several clusters localizing to the bilateral septal nuclei, motor cortex, cingulum, and genu of the corpus callosum (Fig. 3F). Smaller clusters of reduced DAI were identified in various cortical regions with interesting laterality in PME offspring (Fig. 4B). Extensive regions of reduced ODI were observed throughout the brain of PME offspring compared to PSE controls (Fig. 4A). Nearly all regions of the cortex exhibited significantly lower ODI bilaterally including the auditory, visual, retrosplenial, somatosensory, motor, and cingulate cortices as well as adjacent white matter such as the corpus callosum, cingulum, and external capsule (Fig. 4A). There were some limited extensions into the posterior insular cortex and piriform cortex as well (Fig. 4). Several large clusters were identified throughout the dorsal and ventral hippocampus bilaterally as well as connecting white matter including the fornix and hippocampal commissure (Fig. 4A). These significant clusters also extended into the amygdala dorsolaterally and habenula ventromedially. Clusters of reduced ODI were also identified in several thalamic nuclei bilaterally. Lastly, there was extensive involvement of the basal ganglia structures including, large portions of the dorsal striatum and globus pallidus, as well as the claustrum, and adjacent internal capsule.

## Discussion

Using a previously characterized mouse model of PME with well-described impairments in neurobehavioral development, we employed ultra-high field diffusion MRI to examine the long-term impact of PME on brain tissue microstructural organization using a robust, unbiased voxel-based analysis. PME differentially altered microstructure across various cortical and subcortical regions. These results suggest exposure to methadone during pregnancy induces a persistent and complex effect on tissue microstructure, specifically neurite morphology, that remains detectable into early adulthood. The present work represents the first in vivo investigation of tissue microstructure in a translationally relevant model of prenatal opioid exposure, and as such, may provide insights into the mechanisms underlying disrupted cognitive and behavioral development often observed in children with prenatal opioid exposure^57,58^.

The use of both signal representation scheme (DTI) and tissue specific model (NODDI) represents a strength of the present study. The application of DTI allowed us to estimate the degree of sensitivity of PME-induced microstructural alterations and the NODDI model helped us to characterize these changes more specifically. While DTI is generally sensitive to changes in microstructure, its interpretation in regions of the brain with more complex structure such as areas with crossing fibers or where microarchitecture includes a mixture of cell bodies, axonal tracts, and dendrites is not trivial^42,43^. For instance, FA is often referred to as an indicator of white matter integrity, but this is likely too simplistic of a description. In more nuanced and complex pathological states like prenatal opioid exposure where neurons and glia are developing in an environment with constant exogenous opioid exposure, there is likely to be both a direct effect of opioids on neurodevelopment and several compensatory changes which could produce a combination of neuroinflammation, demyelination, neuronal apoptosis, gliosis, increased dendritic arborization, dendritic loss, axonal packing, and/or axonal degeneration creating competing effects on the diffusion tensor in a region of interest^59^. Employing MD, AD, and RD can aid in further description of the diffusion tensor and better assess tissue microstructure. However, NODDI allows for a more specific characterization of microstructure in both grey and white matter, including neurite density and dispersion orientation which provide deeper insight into the effects of various insults on neurite morphology. Furthermore, we utilized the Bingham–NODDI model which better characterizes the anisotropic orientation dispersion of neurites which is a common attribute of neurons in many parts of the brain.

The clusters demonstrating significant differences in DTI and NODDI metrics were localized to several distinct cortical and subcortical brain regions that support findings from other studies of prenatal opioid exposure. Given the large number of brain regions affected, we cannot discuss each individually but can highlight a few with perhaps more relevance to the known neurobehavioral-related alterations in the brains of prenatal opioid exposure based on previous work. Significantly increased MD in the hippocampus is suggestive of reduced packing density or membrane density and could reflect damage to this region in PME offspring. Furthermore, ODI was significantly reduced in nearly the entire hippocampal structure bilaterally which could reflect reduced dendritic arborization in PME offspring. In possible support of this, Oberoi et al. described reduced expression of myelin associated proteins in the hippocampus in rat pups with PME indicative of hypomyelination as a result of PME^60^.

Altered hippocampal microstructure may very well be related to the impairments in various memory tasks that have been studied extensively in the context of prenatal opioid exposure^61,62^. Additionally, significant clusters of diverse microstructural differences were frequently identified in the somatosensory cortex, motor cortex, dorsal striatum, and their respective adjacent white matter. We and others have described delayed development of several sensorimotor-based developmental milestones including the cliff aversion, surface righting, and forelimb grasp task, among others, which suggest opioid exposed offspring may lack the ability to translate multimodal sensory input into complex motor behaviors^21,63–65^. Furthermore, we have also demonstrated that PME disrupts synaptic signaling in the somatosensory cortex, motor cortex and dorsal striatum using brain slice, patch-clamp electrophysiology^21,26,27^. Therefore, these changes in diffusion tensor and neurite dispersion metrics may reflect alterations in synaptic architecture related to our observed differences in synaptic transmission in PME offspring. Lastly, the insular cortex is emerging as an integral hub associated with many psychopathologies^66^, and often exhibits altered functional connectivity in human studies of prenatal substance exposure^16,67,68^. Using a functional connectomics approach, Merhar et al. identified unique connections between the left insular cortex and the right caudate and amygdala in infants with opioids exposure during the entire gestational period^16^. We add to these findings by reporting prenatal opioid exposure disrupts various DTI and NODDI metrics in the anterior and posterior insular cortex suggesting disorganization to insular microstructure and insular neurite morphology in PME offspring.

Mechanistic insights into the impact of opioids on DTI and NODDI metrics is the subject of further investigation. Methadone acts as an agonist at the mu opioid receptor (MORs), which is the canonical opioid receptor associated with the rewarding and analgesic effects of opioids^69,70^. However, endogenous opioid peptides and their respective opioid receptors (including the MOR) can be detected in the mouse brain beginning at embryonic day 11.5 where they serve in an inhibitory role to modulate neuronal proliferation, migration, and differentiation^71–74^. Although other models of prenatal opioid exposure have demonstrated a decrease in neural cell proliferation and cell density in several brain regions^75–78^, our assessments have not revealed such gross changes to neuroanatomy. For instance, there was only a minimal effect of PME on cell density within the somatosensory, motor, and anterior cingulate cortex of male offspring^21^. Furthermore, ultra-high field structural MRI did not reveal any differences in various cortical and subcortical grey matter volumes suggesting the effects of our PME model may impact more terminal process of neurodevelopment such as axonogenesis and dendritogenesis which would not be identified by cell density or volumetric MRI assessments^21^. Indeed, when examining large-scale proteomic and phosphoproteomic data in the dorsal striatum and somatosensory cortex of PME male offspring, we discovered an enrichment in proteins/phosphopeptides associated with the axonal and dendritic cellular compartment and proteins/phosphopeptides which function in axonal guidance, the maintenance of synaptic architecture, and NGF-mediated transcription^26,27^. Similarly, other models of prenatal opioid exposure have identified differences in the expression of various growth factors and their receptors such as NGF and BDNF which are known to be involved in axonal growth and dendritic branching^79–82^. Therefore, it is reasonable to hypothesize that these microstructural changes in the brain of PME offspring result from exposure to methadone during embryogenesis which disrupts normal neurite development that may be normally modulated through the endogenous opioid system.

Alternatively, prenatal opioid exposure may disrupt gliogenesis or interfere with normal neuronal-glial interactions^83,84^. Oligodendrocytes, the myelin producing cells of the CNS, also express opioid receptors and respond to exogenous opioids^83,85^. Prenatal opioid exposure has been shown to disrupt myelin structure and alter the expression of various myelin associated proteins^86–88^. Jantzie et al. examined two white matter regions (the corpus callosum and external capsule) and identified increased RD in early adolescent rat offspring with PME using ex vivo DTI which aligns with our findings of increased RD in a cluster localized to the external capsule. However, their sample sizes were quite small and the gestational exposure to methadone did not begin until embryonic day 16 which differs significantly from our model making direct comparisons challenging^28^. Additionally, formalin fixed tissue (ex-vivo) increases tissue rigidity due to protein cross-linking^89^, changes diffusion profile across tissue^90^ and alters intra/extra cellular compartment composition^91^. Thus, direct comparison between ex vivo and in vivo results are quite subjective. Interestingly, Jantzie et al. also discovered significant increases in brain chemokines/cytokines and altered cortical microglia morphology in rat pups with PME^28^. We and others have discovered effects of opioid exposure on microglia^26,92^. Prenatal oxycodone exposure leads to reduced adolescent microglial phagocytosis of D1 dopamine receptors and subsequently higher D1R density within the nucleus accumbens in adult male offspring suggesting microglial sculpting of neurons is disrupted by opioid exposure^92^. In the somatosensory cortex, we identified a reduction of microglia^26^. This loss of microglia was associated with decreased expression of presynaptic GABAergic markers, increased expression of postsynaptic GABAergic markers, decreased inhibitory synapses in adolescent PME offspring, and a disruption of functional GABAergic synapses that was more prominent in male offspring with PME^26^. It remains possible that microglial-mediated pruning of synapses may be disrupted in PME offspring leading to some of the microstructural changes observed in the present study.

There are some limitations to this work which should be briefly discussed. Given the exploratory nature of the present work, only male offspring were utilized. We note that in our prior behavioral study of sensorimotor development we did not observe many significant sex differences^21^. However, additional work from our laboratory using this model have revealed behavioral, physiological, and biochemical sex differences^25–27^. Future work will need to examine female offspring to confirm findings or explore sex-effects in neuroimaging. We have not observed significant differences in maternal care behaviors between opioid and saline treated dams^21^, but there may be subtle differences in maternal care which we are not able to observe that interact with opioid exposure to produce the findings reported. Animals were lightly anesthetized with isoflurane to facilitate imaging which is a standard approach to small rodent imaging, but it is possible (although unlikely) sedation with isoflurane could influence microstructural measurements. To minimize scanning session time and increase resolution, we only examined regions between the frontal cortex and cerebellum. Future work will need to assess how hindbrain microstructure may be affected.

Overall, this in vivo assessment of microstructural integrity in a translationally relevant mouse model of PME revealed several perturbations to microstructural complexity and neurite organization that persist into early adulthood. These findings deepen the understanding of prenatal opioid exposure on central nervous system development and may also have important clinical implications for the growing population of children with prenatal exposure to opioids.

## Abbreviations

AD: Axial diffusivity
API: Application programming interface
DAI: Dispersion anisotropy index
dMRI: Diffusion magnetic resonance imaging
DTI: Diffusion tensor imaging
DWI: Diffusion weighted imaging
EPI: Echo planar imaging
FA: Fractional anisotropy
GI: Gastrointestinal
JDM: Jacobian determinant map
MD: Mean diffusivity
MRI: Magnetic resonance imaging
MRS: Magnetic resonance spectroscopy
NODDI: Neurite orientation dispersion and density imaging
ODI: Orientation dispersion index
PME: Prenatal methadone exposure
PSE: Prenatal saline exposure
RD: Radial diffusivity
TE: Echo time
TR: Repetition time
VF_IC_: Intracellular volume fraction
VF_ISO_: Volume fraction of isotropic water diffusion
